# The Problem of Auto-Correlation in Parasitology

**DOI:** 10.1371/journal.ppat.1002590

**Published:** 2012-04-12

**Authors:** Laura C. Pollitt, Sarah E. Reece, Nicole Mideo, Daniel H. Nussey, Nick Colegrave

**Affiliations:** 1 Institute of Evolutionary Biology, University of Edinburgh, Edinburgh, School of Biological Sciences, Edinburgh, United Kingdom; 2 Centre for Infectious Disease Dynamics, Pennsylvania State University, University Park, Pennsylvania, United States of America; 3 Centre for Immunity, Infection and Evolution, University of Edinburgh, School of Biological Sciences, Edinburgh, United Kingdom; The Fox Chase Cancer Center, United States of America

## Abstract

Explaining the contribution of host and pathogen factors in driving infection dynamics is a major ambition in parasitology. There is increasing recognition that analyses based on single summary measures of an infection (e.g., peak parasitaemia) do not adequately capture infection dynamics and so, the appropriate use of statistical techniques to analyse dynamics is necessary to understand infections and, ultimately, control parasites. However, the complexities of within-host environments mean that tracking and analysing pathogen dynamics within infections and among hosts poses considerable statistical challenges. Simple statistical models make assumptions that will rarely be satisfied in data collected on host and parasite parameters. In particular, model residuals (unexplained variance in the data) should not be correlated in time or space. Here we demonstrate how failure to account for such correlations can result in incorrect biological inference from statistical analysis. We then show how mixed effects models can be used as a powerful tool to analyse such repeated measures data in the hope that this will encourage better statistical practices in parasitology.

## Mixed Effects Models as Important Tools for Parasitologists

Parasitologists aim to understand the factors that determine the outcome of infections (e.g., host and pathogen genetic effects), and how these factors change in response to a new intervention or other environmental variables. However, infections are complex and dynamic: multiple interacting factors shape parasite traits, and within-host environments vary over time and between different niches [1]–[5]. Studies examining infections as a snapshot in time are consequently likely to miss a large degree of complexity and subtle—yet relevant—variation in patterns, and risk missing confounding effects or reporting misleading results. For example, in genetically mixed infections of human malaria parasites, different strains circulate in the blood at different times during the infection [2], and in the lungs of patients with cystic fibrosis, bacterial species compete, which results in varying relative frequencies over time [1]; therefore, single time points are unlikely to be representative of the infection as a whole. While in some cases this problem could be overcome using traditional statistical tests with summary statistics (e.g., the slope of the relationship between parasite density and time), relationships will often not be linear and will co-vary with multiple variables. These dynamical differences have important consequences for disease severity and, as within-host dynamics also determine transmission, are essential to understanding epidemiology [6]. Tracking and analysing pathogen dynamics across and between infections is consequently an important goal, but the statistical complexities of dealing with such data offer many traps for the unwary.

Recent years have seen the increasing use of statistical tools, such as mixed effects models, which allow researchers to analyse pathogen dynamics within infections while controlling for issues of pseudo-replication arising from repeated measurements on the same host (i.e., time-series data [7]). In addition to enabling statistically rigorous tests of biological hypotheses relating to infection dynamics, these approaches cut the number of animals needed in experiments, reducing financial costs and ethical concerns. Mixed effects models work by fitting fixed effects, random effects, and error terms into the model. Fixed effect terms explain the variation in a response variable (e.g., parasite density) that is due to the treatment or predictor variable of interest (e.g., drug versus no drug, competitor versus no competitor). Random effect terms, which are specific to a particular group of observations (e.g., all the measurements made on one individual or group of individuals), describe the constant deviation from the mean of that individual or group. Finally, error terms describe the variation (the residuals) remaining in the data that is not explained by either the fixed or the random terms [8]–[10]. The use of random effect terms allows researchers to make use of all of the available data points, whilst removing many of the statistical problems associated with repeatedly measuring the same individual. See [7], [11], [12] for discussion of additional benefits of this approach.

## The Problem of Auto-Correlation

**Figure 1:**
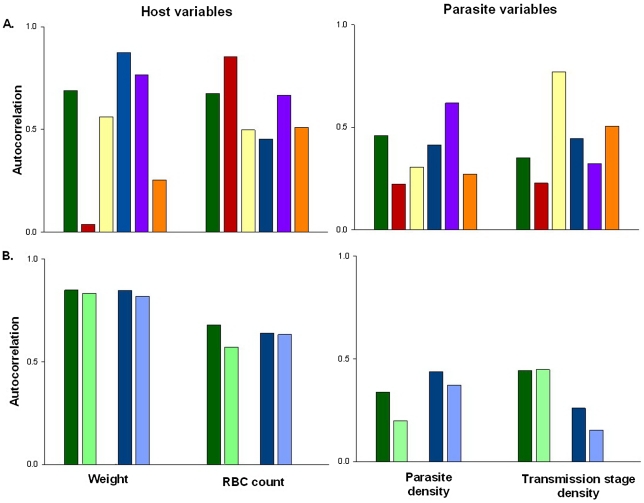
The extent of auto-correlation in experimental rodent malaria infections. Estimates of temporal auto-correlation in key parasite and host traits observed during daily sampling of infections initiated with controlled *Plasmodium chabaudi* parasite doses in mice matched for strain, age, and sex. (A) Data for days 5–15 post infection taken from [16], [17]. Colours represent different wild-type parasite genotypes (green = AS, red = AJ, yellow = ER, blue = DK, purple = CW, orange = CR). (B) Data for days 3–18 post infection taken from [18] in mice with depleted levels of CD4+ T cells (light bars) or unmanipulated immune responses (dark bars) for genotypes AS (green) and DK (blue). These estimates demonstrate how levels of auto-correlation can be both high (up to 87% correlation between residuals on consecutive days) and variable between traits. The implications of this will depend on the analysis performed, but auto-correlation at such high levels has the potential to dramatically increase type 1 error rates (Figure 2).

**Figure 2:**
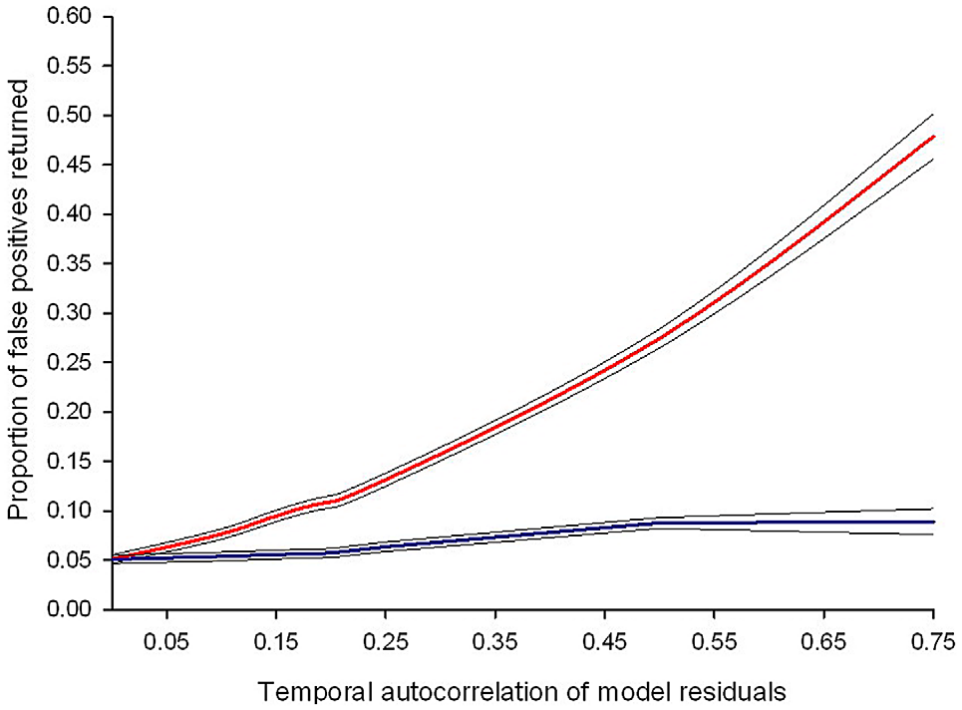
Failing to control for temporal auto-correlation increases type 1 error rates. Simulated data sets with known levels of temporal auto-correlation between residuals (spanning the range observed in published data sets) were generated using R version 2.12.1 (The R Foundation for Statistical Computing; http://www.R-project.org). Auto-correlation is highest between consecutive days and reduces as the duration between data points increases (auto-regressive error structure). The simulated data sets were designed so that any difference in treatment groups was due to chance: if the linear mixed effects model performs correctly it should, by definition, return a *p*-value of ≤0.05, 5% of the time. The red line represents the proportion of false positive results (where a statistically significant difference between treatment groups is wrongly concluded) from linear mixed effects models. The blue line shows the proportion of false positive results if an auto-regressive error structure is included in the model. Values are averaged from a minimum of 10,000 simulated data sets and the black lines show the 95% confidence intervals around the mean.

At the heart of most statistical tests is the “independence assumption”, which states that model residuals should not be correlated in time or space. Studies where individual subjects are measured on multiple occasions (repeated measures studies) contain potential sources of non-independence, which are not present when individuals are only measured once. In particular for time-series data, unmeasured factors can produce correlations in the data (temporal auto-correlation) over days, weeks, or months (i.e., data points adjacent to one another in a time series are more likely to be similar than those further apart). These correlations can be strong and, importantly, may lead to the appearance of spurious patterns in the data (Figures 1 and 2). Failure to account for this temporal non-independence when carrying out an analysis will incorrectly inflate test statistics and can dramatically increase the likelihood of false positives (type 1 errors [13]), where a significant difference between treatment groups is wrongly concluded. Indeed, simulations based on levels of auto-correlation found in real data show that failing to account for it in analyses has the potential to double or even triple the number of false positives (Figure 2).

**Figure 3:**
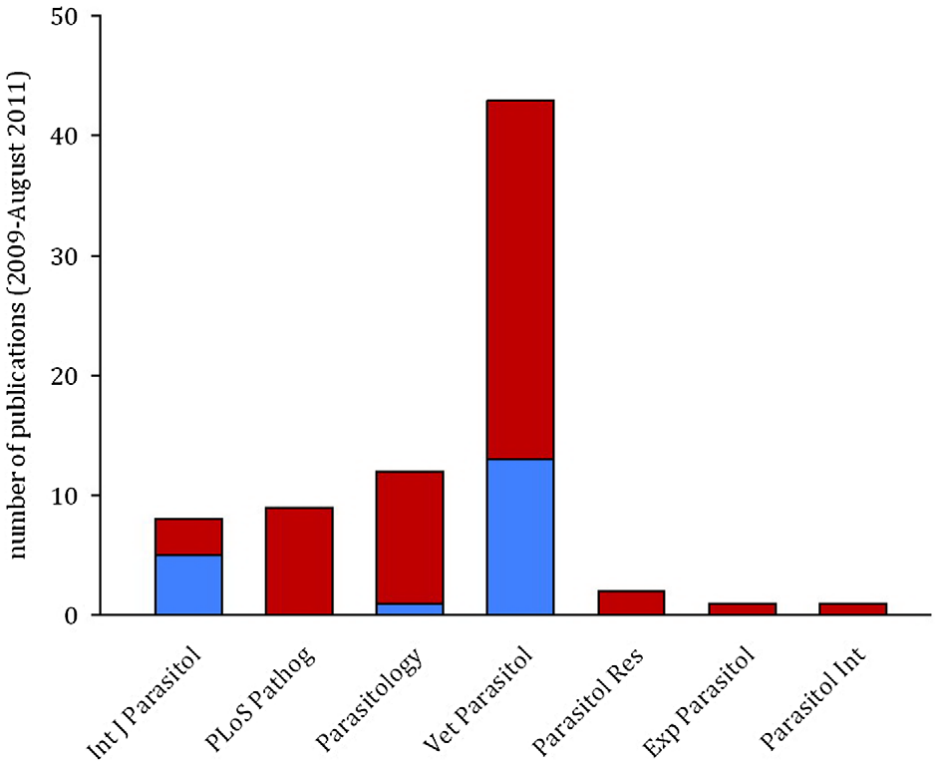
The majority of publications in parasitology do not control for temporal auto-correlation. Bars show the result of a literature search for papers using mixed effects models to analyse time-course data sets in seven parasitology journals from January 2009 to August 2011. Of 76 papers examined, 19 explicitly controlled for temporal auto-correlation (blue), but no controls were mentioned in 56 (red).

There are straightforward solutions to the problems caused by temporal auto-correlation that are routinely used in other biological disciplines, but remain rarely implemented in parasitology. While previous reviews have highlighted the need to track infection dynamics (e.g., [2]) and encouraged the use of statistical models as a valuable tool for parasitologists [7], the importance of meeting the assumptions of these tests has been largely ignored. In a literature search of papers published in the last three years we found that, of 76 papers using mixed effects models to analyse infection data in seven high impact parasitology journals, only 25% of publications explicitly checked and/or controlled for temporal auto-correlation (Figure 3). This indicates a worrying trend and potentially a major problem with the validity of reported findings.

## Controlling for Auto-Correlation

There are various ways in which violations of the auto-correlation assumption can be dealt with. One approach is to make tests more conservative by reducing the *p*-value for which a significant difference is accepted [14], [15]. However, a more elegant (and less arbitrary) alternative is to fit error structures that account for auto-correlation as additional terms in mixed effects models. These error structures describe, and therefore control for, the correlations between residuals at different time points [9]. The simplest form is an auto-regressive error structure, which assumes that the correlation decreases with lag time, e.g., measurements on day 4 of an infection are more similar to those on day 5 than to those on day 20 [9], [13]. Auto-regressive error structures are likely to be common in time-course data [13], are straightforward to understand and implement (see Box 1), and can restore confidence in statistical inference (Figure 2). However, as with all analyses, it is important to consider if this error structure is appropriate for one's own data and whether more complex error structures could potentially provide a better fit (see [9] for examples).

**Box 1. How to Fit Auto-Regressive Error Structures**

When analysing time course data, researchers should apply the following approach: 1) fit grouping variables as a random effect if required, 2) add temporal auto-correlation structure to the model at the appropriate level within the random effects structure, 3) compare the above models to test whether the auto-correlation structure improves the fit, 4) retain the auto-correlation structure if the fit is improved and exclude it if not, 5) ensure the inclusion or exclusion of an auto-correlation structure is reported in the analysis methods or results section of manuscripts.

For example, in the R statistical software package (The R Foundation for Statistical Computing; http://www.R-project.org) a linear mixed effects model asking whether parasite density varies during infections sampled daily in different experimental treatments in mice would be coded as:
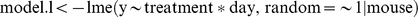
where treatment and day are the fixed factors and the identity of each mouse is a random effect term. The corAR1 correlation function (in the nlme package) will fit an autoregressive error structure and simply requires the model to be specified as:
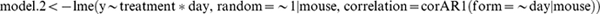
where day is the time covariate and mouse is the grouping factor within the corAR1 function (i.e., because mice are sampled daily, the scale of auto-correlation is day nested within mouse identity). The fit of model.1 and model.2 can then be compared (e.g., using AIC values or a likelihood ratio test). For examples of how to fit alterative error structures and more detailed discussion of running mixed effects models, see [9], [19] for R users and [20] for SAS users.

Advances in statistical methodology should provide important and useful tools for understanding infections and disease in just the same way as do advances in genetic, molecular, and immunological methods. Investing in learning how to effectively use tools, such as mixed effects models, pays by providing robust and novel insight into the roles of hosts and parasites in shaping patterns of disease. However, as with other methodological advances, the improvements to biological understanding they provide depend crucially on them being applied and interpreted correctly. Temporal correlation in time-course data can compromise statistical analyses by increasing the likelihood of false positives, yet this problem has been largely overlooked in parasitology. We strongly support the implementation of more sophisticated statistical analyses in which the assumptions underlying models are fulfilled to safeguard against inaccurate or misleading results and provide a solid foundation from which to progress understanding of disease.
